# Inoculation and phosphorus fertilizer improve food-feed traits of grain legumes in mixed crop-livestock systems of Ethiopia

**DOI:** 10.1016/j.agee.2019.04.014

**Published:** 2019-07-01

**Authors:** Sisay Belete, Melkamu Bezabih, Birhan Abdulkadir, Adugna Tolera, Kindu Mekonnen, Endalkachew Wolde-meskel

**Affiliations:** a Department of Animal Science, Ambo University, Ambo, Ethiopia; b International Livestock Research Institute, Addis Ababa, Ethiopia; c School of Animal and Range Sciences, Hawassa University, Hawassa, Ethiopia; d World Agroforestry Centre (ICRAF), Addis Ababa, Ethiopia

**Keywords:** Crop-livestock, Haulm quality, Rhizobium, Inoculant, Nitrogen fixation, Phosphorus, Legume productivity

## Abstract

Grain legumes play an important role as source of food and feed in smallholder mixed systems. They also contribute to soil fertility improvement through biological nitrogen fixation. Although rhizobium inoculation and phosphorus fertilizer are known to improve grain yield of legumes, information is limited on the effect of this practice on the yield and fodder quality of the haulm. This study was conducted to evaluate the effects of rhizobium inoculation (I) and phosphorus fertilizer (P) on yield and nutritional quality of grains and haulms of grain legumes (faba bean, chickpea, common bean and soybean) on farm across diverse agroecological locations in the Ethiopian highlands. The crops were subjected to four treatments [+I, +P, −I + P and a negative control (-P-I)] at multiple locations on farm during the main cropping season in 2016. Yield data was recorded during grain harvesting, and subsequently representative samples of grains and haulms were collected and analyzed for quality variables. Effects of the treatments were significant (P < 0.05) with 30% increase on grain yield for all studied crops and 28% increase on haulm dry matter yield for faba bean, common bean and soybean. Crude protein (CP) and *in vitro* organic matter digestibility (IVOMD) values of faba bean, common bean and soybean haulms were higher (P < 0.05); and neutral detergent fiber (NDF) and acid detergent fiber (ADF) contents were lower (P < 0.05) for the treatments than the control. The haulm CP content and IVOMD of chickpea also responded positively (P < 0.05) to the treatments. The current results demonstrated the possibility of improving both yield and quality of grains and haulms of grain legumes with the application of efficient rhizobium inocula and P fertilization. This practice offers an opportunity for smallholders in the crop-livestock system to improve the food-feed traits of grain legumes with minimal input and environmental footprint.

## Highlights

• Rhizobium inoculation & P fertilizer on food-feed traits of grain legumes were studied.
• The treatments improved grain and haulm yield of the legumes.
• The fodder quality traits improved concomitantly.
• The technology provides low cost option to improve whole plant productivity.

## 1 Introduction

Food and feed demand in Ethiopia continue to grow at a high rate due to population pressure and high yield gaps both in crop and livestock productivity. Grain legumes are the second most produced crops in the country next to cereals. They are cultivated on more than 1.5 million hectares of land annually, mainly by smallholder farmers in the mixed crop-livestock farming system for food, feed and soil fertility improvement through symbiotic biological nitrogen fixation (CSA, 2015). The role of grain legumes to sustain the smallholder system is becoming indispensable as declining soil fertility continues to be a major challenge in the Ethiopian highlands due to land degradation and erosion (Tesfahunegn et al., 2011; Haileslassie et al., 2005). Nitrogen and P are among the main limiting nutrients in soil systems in Ethiopia that create high yield gaps (Tamene et al., 2017; Wolde-meskel et al., 2018). For instance, Haileslassie et al. (2005) estimated that arable soil nutrients were depleted annually at a rate of 122 kg N ha^−1^, 13 kg P ha^−1^, 82 kg K ha^−1^; while inflow of nutrients from artificial fertilizer application is minimal (less than 20 kg ha^−1^ y^−1^ for N) (CSA, 2015). Under this scenario, a better integration of grain legumes, coupled with improved agronomic practices that enhance biological N fixation, will enable to exploit the full potential of crop legumes in smallholder systems.

The efficiency with which atmospheric N is fixed by legumes as well as the total amount of N incorporated into the soil system can be considerably increased by inoculating the seeds with effective strains of rhizobium (Giller, 2001; Yakubu et al., 2010). However, low P content of the soil may reduce symbiotic efficiency of the legume crop (Yakubu et al., 2010). Studies have shown that inoculation with effective rhizobium strains and small amount of P fertilizer significantly increases grain yields of legumes (Rurangwa et al., 2018; Wolde-meskel et al., 2018). This practice appears to play an important role for the sustainable intensification of smallholder systems in Sub-Saharan Africa because of its potential to enhance both soil fertility and crop yields with low cost (van Heerwaarden et al., 2018).

The livelihood of smallholder farmers in the mixed system is dependent on both crop and livestock production. In most cases, the livestock and crop sub systems have a strong interdependence and complementarities (Getachew et al., 1993; Solomon et al., 2009). Residues from crop cultivation have increasingly become the major source of feed for livestock (Bayush et al., 2008; Malede and Takele, 2014), contributing up to 30–80% of the total feed dry matter available for animals in the highlands of Ethiopia (Africa Rising, 2014). Haulms of grain legumes contain higher crude protein than cereal residues and their contribution to the nutrition of livestock in the smallholder systems is considerable (Lopez et al., 2005; Tolera, 2008). This is especially true during the dry months when green fodder is unavailable and farmers are required to keep strong draft oxen for land preparation at the onset of the rains. In spite of this, agronomic and breeding activities mainly focus on improving grain yields and overlook the importance of residue yield and quality for smallholders.

While inoculation with effective rhizobial inoculant and P fertilizer is demonstrated to increase grain yield in legumes (Deaker et al., 2004; van Heerwaarden et al., 2018; Wolde-meskel et al., 2018), there are few studies on how it affects fodder trait variables (haulm yield and quality) in grain legumes. At the outset of this research, we hypothesized that N fixation due to inoculation and P fertilizer would considerably increase nutrient accumulation in the plant system, which would positively affect the fodder trait of the haulm in terms of yield, crude protein content and digestibility. This will in turn contribute to livestock productivity. To test this hypothesis, an extensive on-farm trial involving rhizobium inoculation and P fertilizer treatments was conducted. Here, we report the effect of the treatments [I, P and/or I + P] on whole plant productivity, grain and haulm yield and haulm fodder quality on four grain legumes namely, chickpea, common bean, faba bean and soybean. The study was conducted across a large number of smallholder farms covering diverse soil fertility and agroecological conditions over four regions in Ethiopia namely, Amhara, Benshangul Gumuz, Oromia and Southern Nations Nationalities and Peoples’ Region. The results are discussed with respect to the potential of the treatments (I, P and/or I + P) to enhance grain-haulm yield and feed quality of legumes, and their implications for wide scale promotion and intensification of crop-livestock systems within smallholder systems.

## 2 Materials and methods

### 2.1 Study sites

**Fig. 1:**
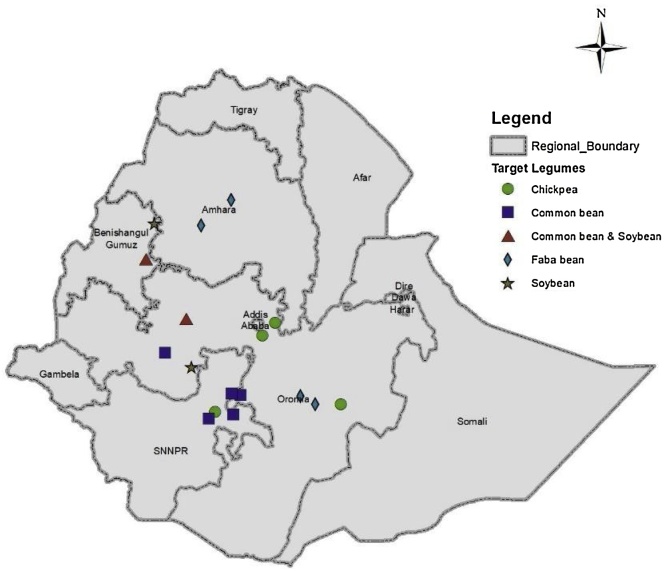
A map showing the locations where the trials were conducted for the different grain legumes in Ethiopia.

The study was conducted in 16 purposely selected districts across four regional states of Ethiopia in the 2016 cropping season. The selection was based on their representativeness and potential for grain legume production. The distribution of the study districts across the four regions is indicated in Fig. 1. The districts have been used as action sites for the N2Africa project, a large scale research-in-development project focusing on putting N fixation to work for smallholder farmers growing legume crops in Africa. Four legume crops of major economic importance were considered for the study and the districts were tagged with the different grain legumes based on the potential of the areas as shown in Table 1 and Fig. 1.

**Table 1:** Altitude, annual mean rainfall, temperature and target crops of the trial districts.

District	Location	Altitude (masl)	Rainfall (mm)	Temperature range (^o^C)	Target legume
Ada’a	8°8’N, 39°1’E	1800	877	12–26	Chickpea
Agarfa	7°3’N, 39°9’E	2050	800	17–27	Faba bean
Bako-tibe	9°1’N, 37°8’E	2160	1100	9–31	Common bean and soybean
Boricha	6°9’N, 38°2’E	1600	850	10–25	Soybean
Damot Gale	6°9’N, 37°8’E	1900	1250	11–26	Chickpea
Dibatie	10°7’N, 36°2’E	1200	800	10–29	Common bean
Farta	11°8’N, 37°9’E	2800	1350	9–25	Faba bean
Gimbichu	8°9’N, 39°1’E	2400	900	13–26	Chickpea
Ginir	7°1’N, 40°6’E	1976	1300	18–27	Chickpea
Halaba	7°3’N, 38°1’E	1700	800	17–20	Common bean
Pawe	11°3’N, 36°4’E	1120	1600	14–32	Common bean and soybean
Shalla	7°2’N, 38°4’E	1650	600	15–25	Common bean
Sinana	7°1’N, 40°1’E	2225	1050	15–18	Faba bean
Sodo-zuria	6°8^’^N, 37°7E	2170	1225	13–26	Common bean
Yilmana Densa	11°2’N, 37°4’E	2240	1000	11–26	Faba bean
Tiroafeta	7°9’N, 37°2’E	2200	1100	13–23	Soybean

### 2.2 Crop type and experimental design

Four grain legumes which are already widely grown in the selected areas were considered. These legumes are faba bean (*Vicia faba*), chickpea (*Cicer arietinum*), soybean (*Glycine max*) and common bean (*Phaseolus vulgaris*). Four to six districts were selected to conduct the experiment using each of the grain legumes. The experiment was designed in such a way that each of the four crops were subjected to four agronomic treatment: 1) a control practice (without inputs of P fertilizer and inoculation, -P-I), 2) applications of P fertilizer only (+P-I), 3) application of rhizobium inoculation only (-P + I), 4) combined application of P fertilizer and inoculants (+P + I).

In each district, four farmer households were selected based on representativeness of the farm and willingness of farmers to allocate land for the on-farm trial. The land was ploughed and prepared to provide a uniform seed bed. At each farm, four 10 × 10m^2^ plots were prepared and the treatments [-P-I (control); +P-I, -P + I, +P + I] were randomly assigned to one of the four plots.

### 2.3 Crop establishment and management

All treatments were applied on each of the farmer’s plots without replication (Ronner et al., 2016; Wolde-meskel et al., 2018). Recommended seed rates and row planting were used for each of the legume crop studied (Ronner and Giller, 2012). Before planting, composite soil samples were collected at a depth of 0–20 cm on at least three sampling points per plot. For the two treatments involving P fertilizer (+P-I and + P + I), a rate of 10 kg P ha^−1^ was used and the fertilizer was applied during planting in the form of diammonium phosphate (DAP). For the rhizobium inoculated treatments (-P + I and + P + I), seeds of faba bean, chickpea, common bean and soybean were inoculated with FB-1035, CP-29, HB-429 and MAR-1495 rhizobium strains, respectively. The rhizobium strains are local isolates already in use for commercial inoculant production by a production company in Ethiopia (MBI, Menagesha Biotech Industry PLC). The strains were sourced from the Ethiopian national research system and passed through all microbiological routines and field trials prior commercial use. In all cases, uninoculated seeds were sown first, followed by the inoculated seeds to avoid cross contamination and the inoculated seeds were planted on the same day they were inoculated. All other crop husbandry practices were similar for the four treatments and according to the recommended agronomic packages. Overall, a total of 104 farmers participated in the study but reliable yield data were obtained from 72 on-farm trials. Yield data (grain and haulm) were recorded and representative samples were taken by treatment and farm across the study districts.

### 2.4 Harvesting, sample collection and yield determination

At physiological maturity, plants from the entire treatment plot area were harvested manually and total above ground biomass yield was recorded for each plot. The harvested plants were threshed separately by treatment plot and grain yield was measured. The haulm weight was determined by subtracting weight of grain from weight of total above ground biomass. Representative samples of grain (150 g) and whole plant haulm composed of stems, leaf and pod husks (750 g) were collected into labelled sample bags for each plot.

### 2.5 Estimation of haulm dry matter yield and harvest index

Subsamples of haulm and grain were dried at 100 °C for 12 h in a forced air oven to determine dry matter (DM) percentages. The grain and haulm dry matter yields (DMY) were calculated by multiplying the weight at harvest by the respective DM%. Dried seeds were counted and harvest index (HI) was calculated as a ratio of total grain yield to total above ground biomass yield.

### 2.6 Laboratory analysis of grain, haulm and soil samples

The chemical composition of haulm and grain samples were analyzed at the Animal Nutrition Laboratory of the International Livestock research Institute, Addis Ababa. The samples used for analysis were dried at 65 °C for 48 h and ground to 1 mm mesh size. Near infrared reflectance spectroscopy (NIRS) prediction was employed for the analysis of both grain and haulm samples using equations calibrated and validated for each crop. The NIRS instrument used was a FOSS Forage Analyzer 5000 with software package WinISI II. Predicted traits were ash, N, neutral detergent fiber (NDF), acid detergent fiber (ADF), acid detergent lignin (ADL) and in vitro organic matter digestibility (IVOMD).

The soil samples were air dried, crushed, and passed through a 2 mm sieve for analysis of pH, cation exchange capacity (CEC), total N, available P and exchangeable cations. All soil analyses were carried out at the International Institute of Tropical Agriculture (IITA) in Ibadan, Nigeria, following standard laboratory procedures: suspension method for pH (1:1 soil to H2O), Kjeldahl method for total N, Mehlich method for available P, Walkley Black method for organic C and ammonium acetate method for exchangeable cations (Na, K, Ca and Mg) and CEC (IITA, 1982).

### 2.7 Statistical analysis

Homogeneity of data was checked in Minitab software using Levene’s test prior to actual analysis of the variance. Then, combined analyses of variance over locations was performed using general linear model (GLM) procedure of SAS 9.1 (SAS Institute Inc., 2004). Probability value of <0.05 was used to declare significant effects of the treatment. In case of significant difference in means, Duncan multiple range test was used to locate mean separation.

## 3 Results

### 3.1 Soil property, grain yield, haulm yield and harvest index

Most of the sampled farms had low level of N and available P, with few exceptions where there was moderate and high level of available P (Table 2). While the variability in N content of the soil among farms remained relatively low (2.5 fold between the min. and max.), the difference in available P was found to be large (18 fold). The majority of the soils were acidic, with some of the farms tending to be alkaline. The CEC was generally moderate for the majority of the soils. Except for faba bean, grain yield increased significantly (P < 0.05) due to the combined application of P fertilizer and rhizobium inoculant compared to the control. Chickpea showed an increase in grain yield of 42%, common bean 23% and soybean 46% (Table 3). The treatments with either P application or rhizobium inoculation resulted in intermediate grain yields. Haulm DM yields significantly increased (P < 0.05) by 27% for faba bean and 45% for soybean due to + P + I treatments compared to the control. With regard to common bean, the haulm yield showed a different pattern, as the difference between + P + I treatment and the control (14%) remained insignificant (P > 0.05), whereas that between + P + I and either -P + I or + P—I were significant (P < 0.05). Chickpea haulm DM yield was found to be similar between the treatments and the control. Harvest indices increased (P < 0.05) by 15% for chickpea and by 6% for common bean compared to the control. On the other hand, the HI of faba bean and soybean remained similar between the treatment and the control groups.

**Table 2:** Results of soil nutrient analysis for samples collected from farms in the study districts.

District	n	pH (H2O)	OC	N	P Meh	Ca	Mg	K	CEC
		(1:1)	(%)	(%)	(ppm)	(cmol+/kg)	(cmol+/kg)	(cmol+/kg)	(cmol+/kg)
Ada’a	9	7.42	1.19	0.12	10.3	28.3	4.27	0.82	28.3
Agarfa	3	5.60	2.37	0.31	6.17	19.5	3.24	0.63	19.5
Bako Tibe	3	5.03	1.88	0.23	1.94	10.4	1.93	0.40	10.4
Boricha	5	5.60	1.67	0.18	22.6	12.2	1.37	1.35	12.2
Damot Gale	10	6.93	2.05	0.26	36.2	25.5	2.98	2.83	25.5
Dibatie	5	6.02	2.02	0.20	21.6	26.8	5.60	0.20	26.8
Farta	3	5.17	1.36	0.16	3.58	13.9	2.17	0.20	13.9
Gimbichu	9	7.54	1.04	0.12	9.84	23.0	2.28	0.57	23.0
Ginir	6	7.02	1.54	0.18	4.60	26.2	3.54	0.85	26.3
Halaba	5	6.04	1.20	0.14	23.9	11.5	1.31	1.82	11.5
Pawe	4	5.93	2.14	0.20	2.59	17.7	5.25	0.42	17.7
Shalla	10	6.31	1.70	0.19	16.1	13.1	1.16	0.96	13.1
Sinana	4	6.80	1.74	0.20	8.48	34.9	3.90	1.69	34.9
Soddo Zuria	3	5.37	1.48	0.19	9.31	8.24	1.06	0.98	8.24
Yilmana Densa	5	5.14	1.69	0.22	2.94	17.8	3.86	0.69	17.8
Tiroafeta	4	5.33	1.95	0.23	9.96	12.0	1.94	0.61	12.0
	Max	7.54	2.37	0.31	36.2	34.9	5.6	2.8	34.9
	Min	5.03	1.04	0.12	1.94	8.24	1.06	0.20	8.24
	Mean	6.08	1.69	0.20	11.9	18.8	2.9	0.9	18.8

OC = organic carbon; P Meh = Phosphorous – Mehlich; CEC = cation exchange capacity.

**Table 3:** Mean grain yield, haulm dry matter yield and harvest index of faba bean (n = 16), chickpea (n = 16), common bean (n = 24) and soybean (n = 16) as affected by phosphorus fertilizer and/or *rhizobium* inoculation across locations.

Crop	Variable	Treatments				*SEM*	*P-value*
		−P−I	+P−I	−P + I	+P + I		
Faba bean							
	Grain yield (t/ha)	2.65^ab^	2.84^a^	2.55^b^	2.87^a^	0.133	*
	Haulm yield (t/ha)	2.85^b^	2.95^b^	3.00^b^	3.61^a^	0.18	*
	Harvest index	0.46	0.46	0.44	0.42	0.013	Ns
Chickpea							
	Grain yield (t/ha)	1.50^b^	1.65^b^	1.98^a^	2.13^a^	0.071	***
	Haulm yield (t/ha)	2.25	2.3	2.47	2.44	0.106	Ns
	Harvest index	0.40^b^	0.40^b^	0.42^ab^	0.46^a^	0.013	*
Common bean							
	Grain yield (t/ha)	1.60^b^	1.74^ab^	1.80^ab^	1.98^a^	0.071	*
	Haulm yield (t/ha)	1.62^ab^	1.54^b^	1.55^b^	1.84^a^	0.066	*
	Harvest index	0.47^b^	0.52^a^	0.51^ab^	0.50^ab^	0.011	*
Soybean							
	Grain yield (t/ha)	1.75^b^	2.10^ab^	2.46^a^	2.56^a^	0.156	*
	Haulm yield (t/ha)	2.12^b^	2.32^b^	3.23^a^	3.07^a^	0.257	**
	Harvest index	0.50	0.52	0.48	0.50	0.033	Ns

^a,b,c^Mean values with different letters of superscript within the rows are significantly different (P<0.05), +P+I= phosphorus fertilizer with inoculation; −P+I= inoculation only; +P−I= phosphorus fertilizer only; −P−I=control; ns=not significant.

Exploration of the soil variables for possible influence on growth and yield of the legume crops revealed no statistically significant relationships. This might be attributed to the extreme variability of the values on soil parameters across locations. However, absolute responses were observed due to the inoculation and P fertilizer treatments at the lower level of soil N (%). For instance, at 38% of the on-farm trials with below 0.19% soil N, average grain yield responses of 500 kg ha^−1^, 230 kg ha^−1^ and 864 kg ha^−1^ were obtained with inoculation, P fertilizer and combined application of both inputs respectively. In addition, negative relationships were noted between available soil P and pH of the soil which was reflected on grain yields. The low soil P availability could be associated with the acidic nature of the soils, especially at the soybean- and faba bean-trial sites. This might also be attributed to P fixation and reduced availability of P within these soils.

### 3.2 Nutritional value of the haulms

The quality of faba bean haulm was significantly improved (P < 0.05) as a result of the combined application of P fertilizer and rhizobium inoculant, with 24% increase in CP, 9% increase in IVOMD and 8% decrease in cell wall fractions (NDF and ADF) (Table 4). Chickpea haulm saw a 31% increase in CP (P < 0.05) and a 4% increase in IVOMD (P < 0.05) over the control. However, the ash and cell wall fractions of chickpea haulm were not affected by the treatments. On the other hand, common bean haulm showed significant (P < 0.05) improvement in all quality variables measured, with 11% increase in ash content, 26% increase in CP, 4% decrease in cell wall fractions and 4% increase in IVOMD over the control. Similarly, soybean haulm quality improved (P < 0.05), with a 44% increase in CP, 5% decrease in cell wall fractions and 3% increase in IVOMD over the control. Generally, treatments involving either P fertilizer or rhizobium inoculum appeared to be intermediate in terms of improving the chemical composition and IVOMD of the four haulm types.

**Table 4:** Mean haulm nutritional value of faba bean (n = 16), chickpea (n = 16), common bean (n = 24) and soybean (n = 16) as affected by *rhizobium* inoculation and/or P fertilizer application across locations.

Crop	Variables	Treatments				SEM	*P-value*
		−P−I	+P−I	−P + I	+P + I		
Faba bean				% DM			
	Ash	6.91	7.78	7.67	7.64	0.399	*ns*
	CP	5.25^b^	6.38^a^	6.45^a^	6.52^a^	0.244	*
	NDF	70.5^a^	64.8^b^	64.9^b^	64.9^b^	1.42	*
	ADF	64.2^a^	58.6^b^	58.6^b^	58.9^b^	1.27	*
	IVOMD	42.9^b^	46.7^a^	46.4^a^	46.9^a^	0.82	*
Chickpea							
	Ash	6.73	6.58	6.91	6.78	0.143	*ns*
	CP	3.31^c^	3.60^b^	3.66^b^	4.32^a^	0.207	***
	NDF	63.2	63.5	62.9	62.1	0.46	*ns*
	ADF	50.8	50.8	50.5	49.2	0.41	*ns*
	IVOMD	45.9^b^	45.8^b^	46.5^ab^	47.7^a^	0.31	**
Common bean							
	Ash	7.63^b^	7.82^b^	8.01^ab^	8.50^a^	0.19	*
	CP	5.94^c^	6.72^b^	6.85^ab^	7.50^a^	0.25	**
	NDF	69.8^a^	69.9^a^	69.0^ab^	67.8^b^	0.52	*
	ADF	56.9^a^	57.2^a^	56.1^ab^	54.9^b^	0.47	*
	IVOMD	55.7^b^	55.8^b^	56.8^ab^	57.8^a^	0.49	*
Soybean							
	Ash	6.06	5.73	5.87	6.06	0.187	*ns*
	CP	4.67^b^	5.30^b^	6.08^a^	4.67^b^	0.327	***
	NDF	76.4^a^	75.5^a^	75.3^ab^	76.4^a^	0.77	*
	ADF	59.8^a^	57.9^b^	57.5^b^	59.8^a^	0.82	*
	IVOMD	49.6^b^	49.4^b^	50.2^ab^	49.6^b^	0.45	*

^a, b, c^Mean values with different letters of superscript within the rows are significantly different P<0.05). +P+I= phosphorus fertilizer with inoculation; −P+I= inoculation only; +P−I= phosphorus fertilizer only; −P−I=control; CP=crude protein; NDF=neutral detergent fiber; ADF=acid detergent fiber; IVOMD=in vitro organic matter digestibility; ns=not significant.

### 3.3 Seed quality of the legume grains

Common bean grain showed improvement (P < 0.05) in all the three quality variables as a result of P application and rhizobium inoculation, with 6% increase in thousand seed weight, 2.2% increase in CP and 1.5% increase in IVOMD (Table 5). Soybean grain was also positively affected (P < 0.01) by the treatments for two of the three variables measured resulting in a 9% increase in CP content and 6% increase in IVOMD over the control. However, chickpea grain did not respond to the treatments for all the variables considered.

**Table 5:** Mean thousand seed weight (TSW), crude protein (CP) and in vitro organic matter digestibility of chickpea (n = 16), common bean (n = 24) and soybean grain (n = 16) as affected by *rhizobium* inoculation and/or P fertilizer application across locations.

Crop	Variables	Treatments				SEM	*P-value*
		−P−I	+P−I	−P+I	+P+I		
Chickpea							
	TSW (g)	278	281	284	284	4.8	***ns***
	CP (% DM)	19.8	19.8	19.7	20.2	0.21	***ns***
	IVOMD (% DM)	71.9	72.0	71.8	72.1	0.18	***ns***
Common bean							
	TSW (g)	199^c^	207^ab^	204^bc^	211^a^	3.4	**
	CP (% DM)	26.9^b^	27.8^a^	26.8^b^	27.5^ab^	0.61	*
	IVOMD (% DM)	82.4^b^	82.6^ab^	82.3^b^	83.2^a^	0.44	*
Soybean							
	TSW (gm)	137	146	146	143	2.6	***ns***
	CP (% DM)	39.3^b^	40.7^b^	43.1^a^	42.9^a^	0.5	**
	IVOMD (% DM)	73.4^b^	75.1^b^	78.3^a^	78.1^a^	0.62	**

^a, b, c^Mean values with different letters of superscript within the rows are significantly different (P<0.05). +P+I= phosphorus fertilizer with inoculation; −P+I= inoculation only; +P−I= phosphorus fertilizer only; −P−I=control; TSW=thousand seed weight; CP=crude protein; IVOMD=in vitro organic matter digestibility; DM=dry matter; ns=not significant.

### 3.4 Effects of treatments on N uptake by the legumes

The N uptake in the grain and the haulm consistently improved with the fertility treatments (Table 6). On average, the N uptake due to inoculation and P application increased by 65% (P < 0.05) in the haulm and by 44% (P < 0.05) in the grain over the control. Out of the three grain legumes, soybean showed the highest response in N uptake, with 106% in the haulm and 60% in the grain. Despite increases in N uptake, the N harvest index (the ratio of N in the grain to total N uptake) remained similar across the treatments and the control.

**Table 6:** Nitrogen uptake (kg/ha) by the grain legumes as affected by rhizobium inoculation and/or P fertilizer application across locations.

Crop	Variables	Treatments				P value
		−P−I	+P−I	−P + I	+P + I	
Chickpea	Haulm N (kg/ha)	11.9^c^	13.2^bc^	14.5^b^	16.9^a^	*
	Grain N (kg/ha)	47.5^c^	52.3^c^	62.4^ab^	68.8^a^	*
	N HI	0.80	0.80	0.81	0.80	ns
Common bean	Haulm N (kg/ha)	15.4^b^	16.6^b^	17.0^ab^	22.1^a^	**
	Grain N (kg/ha)	68.9^b^	77.4^b^	77.2^b^	87.1^a^	*
	N HI	0.82	0.82	0.82	0.80	ns
Soybean	Haulm N (kg/ha)	15.8^b^	19.7^b^	31.4^a^	33.1^a^	***
	Grain N (kg/ha)	110^c^	137^b^	170^a^	176^a^	***
	N HI	0.87	0.87	0.84	0.84	ns

^a,b,c^Values along a row with different superscripts are significantly different (P<0.05). P+I= phosphorus fertilizer with inoculation; −P+I= inoculation only; +P−I= phosphorus fertilizer only; −P−I=control; HI=harvest index.

## 4 Discussion

Low soil fertility has long been identified as a major constraint leading to high yield gaps (Tamene et al., 2017). The soil nutrient analysis results in the present study also indicate that the soils are too low in N and P to support optimal crop growth (Hazelton and Murphy, 2007). In this respect, legumes play a vital role in smallholder systems due to N input into the soil through biological atmospheric N_2_ fixation. The overall efficiency of atmospheric N_2_ fixation is determined with the presence of effective soil rhizobium bacteria population and plant available P. Although numerous reports showed the positive effects of rhizobium inoculation and P fertilizer on grain yield of legumes, few studies have investigated as to how this agronomic practice affects fodder traits of the legumes, which in turn affects the crop-livestock system. This study involved on-farm trials across diverse agroecologies and soil types to generate practical evidence on the effects of rhizobium inoculation and P fertilizer application on yield and nutritive values of grain and haulm of faba bean, chickpea, common bean and soybean.

### 4.1 Effects of rhizobium inoculation and P fertilizer on yield variables

The increased grain yield in the present study agrees with earlier findings where a yield increase ranging from 16 to 100% has been reported due to rhizobium inoculation and P application (Ibsa, 2013; Tagore et al., 2013; Wolde-meskel et al., 2018). The average grain yield improvement in the present study (30%) appears to be considerable in view of its potential to increase land productivity with limited capital inputs in the smallholder system. Moreover, the significant improvement in haulm DM yield of all the studied crops (except chickpea) shows that the treatments favored whole plant growth, which translates into higher haulm yield. In earlier reports, chickpea responded positively in terms of haulm DM yield due to the application of similar treatments (Tagore et al., 2013). Lack of significant difference in chickpea haulm yield in the present observation might be attributed to the leaf shedding nature of the crop at maturity. Biomass yield measurement toward the end of the vegetative stage would provide the true response of chickpea to the treatments in haulm yield. Generally, in the present experiment, the application of P fertilizer and seed inoculation with effective rhizobium strains have positively affected the nodulation and vegetative growth of the plants, which ultimately resulted in increased yield performance.

### 4.2 Effect of rhizobium inoculation and P fertilizer on nutritional value of the haulm

The present study showed that all nutritional quality indicators of the legume haulms were significantly affected by the treatments (Table 2). This result appears to be consistent with published reports (Habbasha et al., 2007; Ibsa, 2013; Tagore et al., 2013). In the present study, the CP content was increased in haulms by 25% in faba bean, 31% in chickpea, 26% in common bean and 44% in soybean over the control as a result of the combined application of rhizobium inoculation and P fertilizer. The improvement in CP content was associated with a decrease in cell wall fractions, which shows an improvement in overall nutritional quality. This was evident from the consistent improvement in IVOMD from 1% in soybean haulm to 4% in faba bean haulm (Table 4). Earlier assessments have indicated that a 1% increase in digestibility of crop residues would result in an increase in animal performance (milk, meat and draft power outputs) in the range of 6–8% (Kristjianson and Zerbini, 1999). Theoretically, this means the improvement in haulm nutritive value in the present study can be translated to 6–24% increase in animal performance in the smallholder systems. Given the importance of livestock in the crop-livestock system and the role of crop residues as livestock feed (Duncan et al., 2016) application of soil fertility treatments tested in this study appear very important to enhance both crop and livestock productivity. In addition to the treatment effects, inherent differences between the grain legumes in haulm nutritive quality traits were visible. For instance, soybean haulm on average showed a 2–3% increase in IVOMD compared to the other legumes. These quality and yield differences would be used as input in deciding which crop and type of management to apply in the context of mixed crop-livestock systems.

### 4.3 Effects of rhizobium inoculation and P fertilizer on grain quality variables

The present study indicated that in addition to positive effects on grain yield, there was a significant improvement on grain quality in terms of thousand seed weight, CP and IVOMD for common bean and soybean, although chickpea grain remained unaffected by the treatments. This has an important economic significance as the treatments have improved grain yield in all cases and quality in two of the three legumes. The concomitant improvement in yield and quality of the grain legumes shows the favorable condition created for the crops to assimilate sufficient amount of N and other nutrients for growth and grain filling. Inoculation and P application appeared to have improved root nodulation and functioning, increasing the efficiency of atmospheric N_2_ fixation, which in turn is used for the synthesis of crude protein (Ayub et al., 2012). The importance of P in the production of protein, phospholipids and phytin in legume grains is well documented (Rahman et al., 2008). With regard to N, for instance, the increase in grain yield would trigger a higher demand for post-anthesis N relocation to meet requirements for grain filling. The fact that in the present study both grain yield and grain N concentration increased due to inoculation and P application suggests that the treatments improved availability of N in the soil system for assimilation and improved performance of the legume crops. While the total N uptake of the grain legumes increased significantly with the treatments, the N harvest index remained similar confirming absence of haulm quality compromise (eg. CP content) with increasing yield (Table 6). In practical terms, as smallholder farmers are usually constrained with capital input to improve their land productivity, the present results offer alternative low-cost and low-input options to sustainably increase yield performances of grain legumes. Detailed analysis of economic and environmental benefits that takes into account yield and quality improvements of these agronomic practices warrant further investigations.

## 5 Conclusion

Rhizobium inoculation and P fertilization significantly improved grain yield in all crops and haulm yield of all crops except chickpea. The soil fertility treatments also improved the protein content of the haulm, reduced the cell wall fractions and increased IVOMD. Similarly, except in chickpea, the treatments enhanced food values of the grains by improving the CP and IVOMD. The results show that rhizobium inoculation and P fertilization can be used to enhance the whole plant value of grain legumes in smallholder mixed crop-livestock systems.

## References

[bib0005] Africa Rising (2014). Africa Research in Sustainable Intensification for the Next Generation Ethiopian Highlands Project Technical Report.

[bib0010] Ayub M., Nadeem M.A., Mohammad N., Tahir M., Tariq M., Ahmad W. (2012). Effect of different levels of P and K on growth, forage yield and quality of cluster bean (*Cyamopsis Tetragonolobus* l.). J. Anim. Plant Sci..

[bib0015] Bayush T., Adugna T., Berg T. (2008). Livestock production and feed resource constraints in Akaki and Lume districts, central Ethiopia. Outlook Agric..

[bib0020] CSA (2015). Central Statistical Agency of Ethiopia. National Statistics Abstracts.

[bib0025] Deaker R., Roughley R.J., Kennedy I.R. (2004). Legume seed inoculation technology: a review. Soil Biol. Biochem..

[bib0030] Duncan A.J., Bachewe F., Mekonnen K., Valbuena D., Rachier G., Lule D., Bahta M., Erenstein O. (2016). Crop residue allocation to livestock feed, soil improvement and other uses along a productivity gradient in Eastern Africa. Agric. Ecosyst. Environ..

[bib0035] Getachew A., Zerbini E., Abate T. (1993). Crop-livestock interaction and implications for animal traction research in the Ethiopian highlands. Proc. 4th National Livestock Improvement Conference.

[bib0040] Giller K.E. (2001). Nitrogen Fixation in Tropical Cropping Systems.

[bib0045] Habbasha E., Hozayn M., Khalafallah M.A. (2007). Integration effect between phosphorus levels and bio-fertilizers on quality and quantity yield of faba bean (*Vicia faba L.)* in newly cultivated sandy soils. Res. J. Agric. Biol. Sci..

[bib0050] Haileslassie A., Priess J., Veldkamp E., Teketay D., Lesschen J.P. (2005). Assessment of soil nutrient depletion and its spatial variability on smallholders’ mixed farming systems in Ethiopia using partial versus full nutrient balances. Agric. Ecosyst. Environ..

[bib0055] Hazelton P., Murphy B. (2007). Interpreting Soil Test Results: What Do All the Numbers Mean?.

[bib0060] Ibsa A. (2013). Agronomic and Symbiotic Characteristics of Chickpea, *Cicer arietinum* (L.), As Influenced by Rhizobium Inoculation and Phosphorus Fertilization Under Farming Systems of Wolaita Area, Ethiopia.

[bib0065] IITA (1982). IITA Manual Series No. 7: Automated and Semi-Automated Methods of Soil and Plant Analysis.

[bib0070] Kristjianson P.M., Zerbini E. (1999). Genetic enhancement of sorghum and millet residues fed to ruminants: an *ex-ante* assessment of returns to research.

[bib0075] Lopez S., David R.D., Javier F.G., Dhanoa M.S., Dijkstra J., James F. (2005). Assessment of nutritive value of cereal and legume straws based on chemical composition and *in vitro* digestibility. J. Sci. Food Agric..

[bib0080] Malede B., Takele A. (2014). Livestock feed resources assessment, constraints and improvement strategies in Ethiopia. Middle-East J. Sci. Res..

[bib0085] Rahman M.M., Bhuiyan M.M.H., Sutradhar G.N.C., Paul A.K. (2008). Effect of phosphorus molybdenum and rhizobium inoculation on yield and yield attributes of mungbean. Int. J. sust. crop prod..

[bib0090] Ronner E., Giller K.E. (2012). Background Information on Agronomy, Farming Systems and Ongoing Projects on Grain Legumes in Ethiopia. arxiv:/www.N2Africa.org.

[bib0095] Ronner E., Franke A.C., Vanlauwe B., Dianda M., Edeh E., Ukem B., Bala A., van Heerwaarden J., Giller K.E. (2016). Understanding variability in soybean yield and response to P-fertilizer and rhizobium inoculants on farmers’ fields in northern Nigeria. Field Crops Res..

[bib0100] Rurangwa E., Vanlauwe B., Giller K.E. (2018). Benefits of inoculation, P fertilizer and manure on yields of common bean and soybean also increase yield of subsequent maize. Agric. Ecosyst. Environ..

[bib0105] SAS Institute Inc (2004). SAS/STAT® 9.1 User’s Guide. Cary.

[bib0110] Solomon B., Solomon M., Alemu Y. (2009). The interdependence of crop-livestock production sectors: the case of Sinana-Dinsho district in Bale highlands of Ethiopia. Agric. Trop. Subtrop..

[bib0115] Tagore G.S., Namdeo S.L., Sharma S.K., Narendra K. (2013). Effect of rhizobium and phosphate solubilizing bacterial inoculants on symbiotic traits, nodule leghemoglobin, and yield of chickpea genotypes. Int. J. Agron..

[bib0120] Tamene L., Amede T., Kihara J., Tibebe D., Schulz S. (2017). A Review of Soil Fertility Management and Crop Response to Fertilizer Application in Ethiopia: Towards Development of Site- and Context-specific Fertilizer Recommendation. CIAT Publication No. 443. http://hdl.handle.net/10568/82996.

[bib0125] Tesfahunegn G.B., Tamene L., Vlek P.L.G. (2011). A participatory soil quality assessment in Northern Ethiopia’s Mai-Negus catchment. Catena.

[bib0130] Tolera A. (2008). Feed Resources and Feeding Management: a Manual for Feedlot Operators and Development Workers. Ethiopia Sanitary & Phytosanitary Standards and Livestock & Meat Marketing Program (SPS-LMM) Texas Agricultural Experiment Station.

[bib0135] Van Heerwaarden J., Baijukya F., Kyei-Boahen S., Adjei-Nsiah S., Ebanyat P., Kamai N., Wolde-meskel E., Kanampiu F., Vanlauwe B., Giller K. (2018). Soyabean response to rhizobium inoculation across sub-Saharan Africa: patterns of variation and the role of promiscuity. Agric. Ecosyst. Environ..

[bib0140] Wolde-meskel E., van Heerwaarden J., Abdulkadir B., Kassa S., Aliyi I., Degefu T., Wakweya K., Kanampiu F., Giller K.E. (2018). Additive yield response of chickpea (*Cicer arietinum L*.) to rhizobium inoculation and phosphorus fertilizer across smallholder farms in Ethiopia. Agric. Ecosyst. Environ..

[bib0145] Yakubu H., Kwari J.D., Sandabe M.K. (2010). Effect of phosphorus fertilizer on nitrogen fixation by some grain legume varieties in Sudano-Sahelian zone of north eastern Nigeria. Nigerian J. Basic Appl. Sci..

